# The Association Between Anti-Neoplastic Effects of Curcumin and Urogenital Cancers: A Systematic Review

**DOI:** 10.1155/2024/9347381

**Published:** 2024-10-15

**Authors:** Sadegh Mazaheri-Tehrani, Shiva Rouzbahani, Motahar Heidari-Beni

**Affiliations:** ^1^Department of Pediatrics, Child Growth and Development Research Center, Research Institute for Primordial Prevention of Non-Communicable Disease, Isfahan University of Medical Sciences, Isfahan, Iran; ^2^Student Research Committee, Isfahan University of Medical Sciences, Isfahan, Iran; ^3^Bascom Palmer Eye Institute, Miller School of Medicine, University of Miami, Miami, Florida, USA; ^4^Department of Nutrition, Child Growth and Development Research Center, Research Institute for Primordial Prevention of Non-Communicable Disease, Isfahan University of Medical Sciences, Isfahan, Iran

**Keywords:** curcumin, microRNAs, neoplasms, urogenital neoplasms

## Abstract

**Background:** Curcumin is a polyphenol compound with anticancer effects. We aimed to review the anti-neoplastic effects of curcumin on urogenital cancers, by regulating different microRNA expressions.

**Methods:** A systematic search was conducted in Medline (PubMed), Embase, Scopus, and Web of Science up to the end of August 2024. All English, in vitro, and observational studies that evaluated the effect of curcumin on preventing or treating urogenital cancers through its impact on microRNA expression were included. In vivo or silico studies were excluded.

**Result:** A total of 2549 records were found. Finally, 25 studies were included. Twelve studies assessed the effect of curcumin on prostate cancer, six studies on ovarian cancer, three studies on cervical cancer, three studies on bladder cancer, and one study on renal cancer. MicroRNAs are small noncoding RNAs that regulate the post-transcriptional pathways. They possess pivotal roles in different fundamental mechanisms in cells such as differentiation, migration, apoptosis, and proliferation. Curcumin exerts its anticancer effects on urogenital neoplasms by upregulating tumor suppressor microRNAs (miR-143, miR-145, miR-Let-7, miR-101, miR-3127, miR-3178, miR-1275, miR-3198, miR-1908, miR-770, miR-1247, miR-411, miR-34a, miR-383, miR-708, miR-483, miR-199a, miR-335, miR-503, miR-10b, miR-551a, miR-9, miR-203, miR-7110, miR-29b, and miR-126) and downregulating oncogenic microRNAs (miR-21, miR-210, miR-382, miR-654, miR-494, miR-193b, miR-671, miR-222, miR-23b, miR-664, miR-183, miR-214, miR-320a, miR-23a, miR-30a, miR-320d, miR-1285, miR-32, miR-181a, miR-205, miR-216a, miR-1246, and miR-106b).

**Conclusion:** Cell proliferation is inhibited, and cell apoptosis is induced by curcumin in different urogenital cancers through suppressing oncogenic microRNAs or provoking tumor suppressor microRNAs.

## 1. Introduction

Prostate, bladder, kidney, testis, ovary, uterus, and cervix cancers are the most common types of urogenital neoplasms [1]. Based on global cancer statistics, approximately 20.3% of all cancers and 14.7% of all deaths due to malignancies are related to urogenital cancers [2]. Primary prevention, early diagnosis, and reducing exposure to carcinogens decrease mortality due to cancer. [3, 4].

Genetic and epigenetic alterations can affect cancer initiation and progression through activation of oncogenic genes or inactivation of tumor suppressor genes [5]. Findings have shown some beneficial effects of herbal medicine on the cancer process by modulating different epigenetic processes [6]. Various in vitro and in vivo studies have evaluated the efficiency of dietary polyphenols in the management of cancers [7, 8]. Curcumin (also known as diferuloylmethane) is a polyphenol that is extracted from turmeric (*Curcuma longa*) [9, 10]. Curcumin has different therapeutic effects on chronic diseases such as metabolic syndrome, diabetes, arthritis, inflammatory bowel diseases, and cardiovascular diseases [11, 12]. It has antioxidant and anti-inflammatory properties [13]. In addition, it has antitumor, antimetastatic, and even apoptotic functions in cancer cells [14].

Recently, it has been revealed that curcumin affects cancer progression by different epigenetic mechanisms [15, 16], including DNA methylation [17], histone modification [18], and microRNA expression [19]. MicroRNAs are noncoding RNAs, playing a pivotal role in post-transcriptional pathways, by inhibiting messenger RNAs (mRNA) [20]. The effect of curcumin on microRNA expression and neoplasm progression has been explored widely with inconsistent results [21]. There is no systematic review related to curcumin and urogenital cancers, despite conflicting results [14, 22–24]. So, we aimed to systematically review the association between curcumin and urogenital cancers, through the effect of curcumin on microRNA expression.

## 2. Materials and Methods

### 2.1. Search Strategy

This study was conducted based on the Preferred Reporting Items for Systematic Reviews and Meta-Analysis (PRISMA) 2020 guideline [25]. The protocol of this review is available on PROSPERO (CRD42021223856). A systematic literature review was performed in Scopus, Medline (PubMed), Web of Science, and Embase up to end of August 2024 using the following search string: ((“curcumin” OR “curcuminoids” OR “curcuma” OR “turmeric” OR “diferuloylmethane” OR “turmeric Yellow”) AND (“microRNA” OR “microRNAs” OR “miRNA” OR “miRNAs”) AND (“neoplasm” OR “cancer” OR “malignancy” OR “carcinoma” OR “tumor”)). Two independent reviewers (S.M.T. and M.H.B.) screened the records based on title, abstract, and full text. In addition, relevant review papers were checked to find undetected appropriate articles.

### 2.2. Inclusion and Exclusion Criteria

All English, in vitro, and observational studies that evaluated the effect of curcumin or its analogs on preventing or treating urogenital cancers (including prostate, ovarian, cervical, bladder, and renal cancer) through its effect on microRNA expression were included. In vivo or in silico studies were excluded.

The PICO (population, intervention, comparison, and outcome) of the study was different neoplastic cell lines (population), curcumin or its analogs (intervention), no intervention or intervention except curcumin (comparison), and modulation of proliferation, apoptosis, and migration of neoplastic cells (outcome).

### 2.3. Data Extraction

Two independent reviewers (S.M.T. and M.H.B.) performed data extraction from included articles. The first author's name, study design, publication date, study participants, sample size, gender, age range, type of microRNA, type of neoplasm, and a summary of the main findings were extracted.

## 3. Results

### 3.1. Study Selection Process

**Figure 1:**
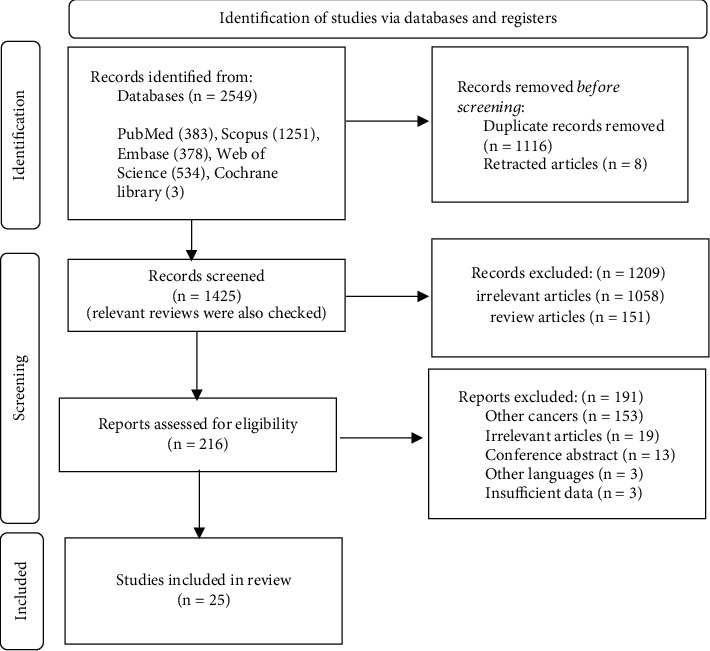
PRISMA flow diagram for the study selection process.

A total of 2549 records were found with systematic search. After duplication removal, 1425 articles remained for screening. Subsequently, 216 papers met the criteria for further assessment via full text. Finally, 25 relevant studies were selected to assess the effect of curcumin on urogenital neoplasms through different microRNA expression.  Figure 1. Shows the study selection process.

### 3.2. Study Characteristics

All included studies were in vitro investigations. In all studies, one group of cells received either curcumin or one type of its analogs. Twenty-two studies used curcumin (diferuloylmethane), one study used 3, 4-difluorobenzylidene curcumin (CDF) [26], one study used demethoxycurcumin [27], one study used nanocurcumin (poly lactic co glycolic acid (PLGA) curcumin) [28], and one study used EF-24 [29] (a synthetic analog of curcumin). As a vehicle control, most of the studies utilized dimethyl sulfoxide (DMSO). Twelve studies assessed the effect of curcumin on prostate cancer, six studies on ovarian cancer, three studies on cervical cancer, three studies on bladder cancer, and one study on renal cancer. Furthermore, the effect of curcumin on the expression of different microRNAs was evaluated relative to endogenous controls such as RUN48 and U6 (small nucleolar RNAs). Some studies also assessed the effect of different doses of curcumin on microRNA expression.

Most of the included studies (16 papers) were conducted in China. Four studies were in the United States, three studies in Iran, and two studies in India.  Table 1 summarizes the features of the included studies.

### 3.3. Prostate Cancer

Studies used different human prostate cancer cell lines including PC-3 [26, 30–35], DU145 [28–31, 34, 36, 37], LNCaP [34, 35], CD44+/CD133+ HuPCaSCs [38], C4-2 [28], and 22RV1 [30, 37].

The effect of curcumin on the expression of different microRNAs associated with prostate cancer has been evaluated. The following microRNAs were reported to be downregulated: miR-21 (two studies), miR-210 (two studies), miR-382, miR-654-3p, miR-494, miR-193b, miR-671, miR-210, miR-222, miR-23b, miR-664, miR-183, and the following microRNAs were upregulated: miR-143 (two studies), miR-145 (two studies), miR-Let-7c, miR-Let-7d, miR-101, miR-145, miR-3127, miR-3178, miR-1275, miR-3198, miR-1908, miR-770-5p, miR-1247, miR-411, miR-34a, miR-30a-5p, miR-383, miR-708, and miR-483-3p. The expression of the following microRNAs did not change: miR-100, miR-126, miR-181a, miR-200a, and miR-148a.

### 3.4. Ovarian Cancer

The human ovarian cancer cell lines SKOV3 [27, 39–41], PA1 [42], A2780 [41, 42], and A2780 cisplatin-resistant subline (A2780cp) [43] were used in the included studies.

The following microRNAs were downregulated using curcumin: miR-214, miR-320a, miR-21, miR-23a, miR-30a, miR-320d, miR-1285, miR-32, miR-181a, miR-205, miR-216a and the following microRNAs were upregulated: miR-551a, miR-9, miR-34a, miR-199a, miR-335, miR-503, and miR-10b. miR-124 did not significantly change with curcumin therapy. Some microRNAs including miR-122b, miR-129, and miR-182 showed controversial results in different cell lines.

### 3.5. Cervical Cancer

Different human cervical cancer lines including HeLa [44], SiHa [44], HPV-16-positive SiHa [23], and HPV-16-positive Ca Ski [22] (adhesive epithelial cell line) were utilized in the included research.

Curcumin resulted in the upregulation of miR-29b and miR-126, while there is conflicting evidence regarding its effect on miR-21. A study on HPV-16 positive Ca Ski cell line indicated no significant change in miR-21 and miR-210 expression after 48 h of using 80 *μ*M curcumin [22]. However, another study on the HPV-16-positive SiHa cell line indicated a decrease in miR-21 expression in a dose-dependent manner of curcumin therapy [23].

### 3.6. Bladder Cancer

Bladder cancer cell lines including T24 [24, 45, 46], J82 [45, 46], TCCSUP [45, 46], and HT-1376 (human grade 3 urinary bladder carcinoma) [24] were used in the included papers.

miR-203, miR-7110, and miR-let-7c were upregulated and miR-1246 was downregulated after using curcumin. There is a conflicting result for the effect of curcumin on miR-7641 expression. Wang et al. [46] demonstrated a significant downregulation in miR-7641 expression in all T24, J82, and TCCSUP cell lines after 24 h of 20 *μ*M curcumin usage. While Xu et al. [24] found a significant upregulation in miR-7641 expression in the T24 cell line after 72 h of 10 *μ*M curcumin usage.

### 3.7. Renal Cancer

There is only one study on renal cancers [47], which used human renal cell carcinoma cell lines ACHN and Caki-2. There is a significant reduction in miR-106-b expression after curcumin usage.

## 4. Discussion

**Figure 2:**
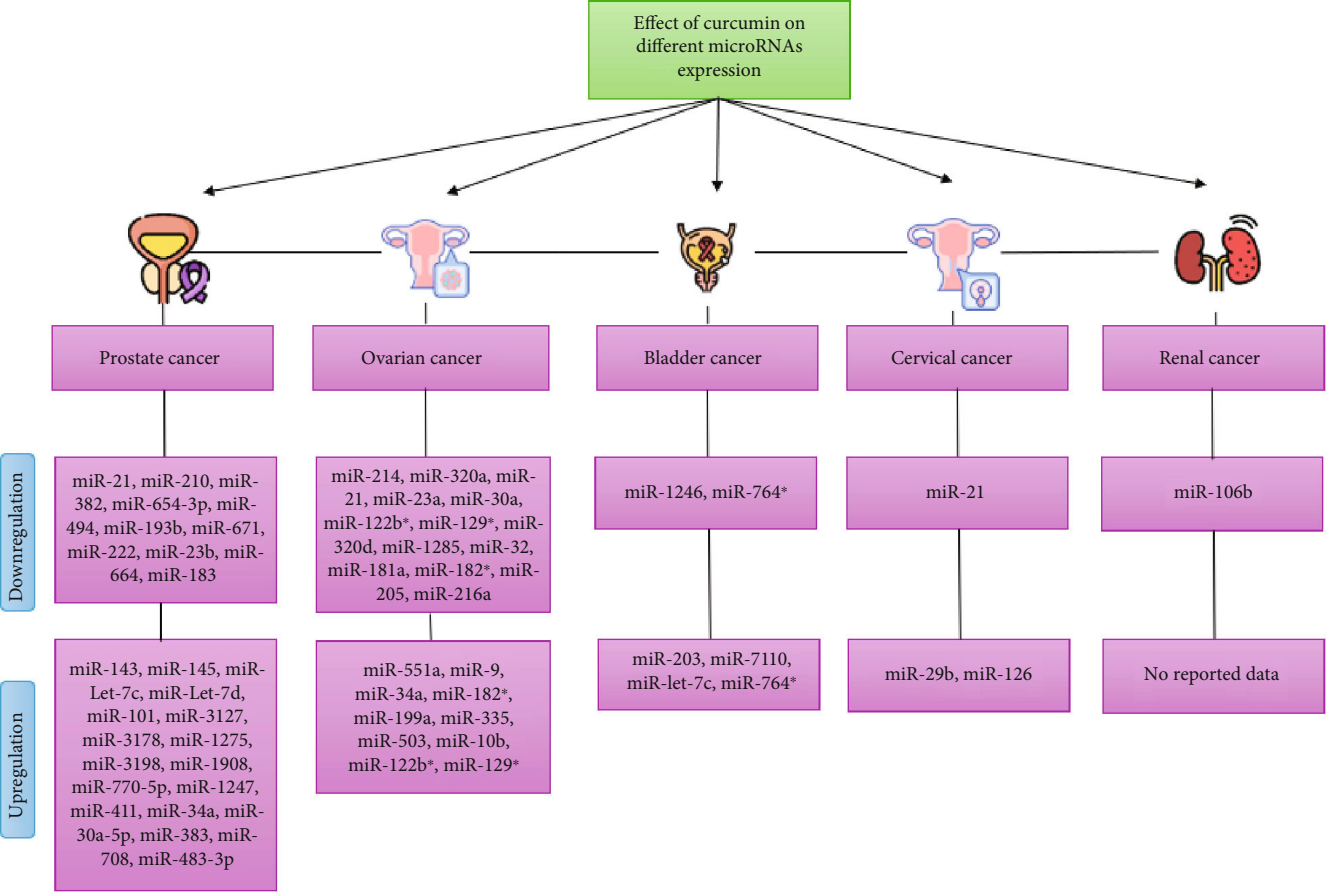
Summary of the effect of curcumin on different microRNA expressions. ^∗^Controversial results.

Results of studies showed that curcumin usage can affect urogenital cancers by alteration in different microRNAs expression (Figure 2). One of the most reviewed mechanisms that transform normal cells into neoplasms and tumors is through alterations in gene expression. There are various regulators associated with the neoplasms [48]. MicroRNAs are important regulators in the post-transcriptional pathways [49]. They are small noncoding RNAs that play a pivotal role in different fundamental pathways in cells such as differentiation, migration, apoptosis, and proliferation [50]. They exert their effect by blocking mRNA translation. MicroRNAs are classified into two groups, based on their role in neoplasms: oncogenic microRNAs and tumor suppressor microRNAs [51]. Oncogenic microRNAs contribute to the initiation, progression, and invasion of tumors by inhibiting tumor suppressor genes and increasing the expression of oncogenic genes [52]. Tumor suppressor microRNAs play a role in the inhibition of oncogenes [53].

In addition to the role of microRNAs in the initiation and development of cancers, they are also markers of responding to anticancer therapy and drug resistance [54]. Recently, many microRNAs have been developed for therapeutic strategies. Some microRNAs lead to replacement or overexpression of tumor suppressor microRNAs or inhibit oncogenic microRNAs [55].

Recently different natural products have been used for the prevention or treatment of cancers, due to their lower price, safety profile, and multitargeting ability in comparison to conventional therapies [56]. Bioactive compounds such as curcumin, resveratrol, lignan, quercetin, and tannins have anticancer effects [57]. Curcumin is one of the well-reviewed compounds in cancer treatment. Curcumin affects cell cycle progression and suppresses proliferation. In addition, it decreases reactive oxygen species (ROSs), inhibits the cell cycle through arresting in the S phase, and directly affects different signaling ways [58–60]. Moreover, recent studies have focused on the regulation of curcumin on different microRNAs expression, as another pathway for inhibiting cell cycle progression and proliferation [9]. Curcumin could affect the metastasis of different neoplasms through targeting metastamirs. [58]. Metastamirs describe microRNAs that are involved in the metastasis of tumors. They are divided into antimetastatic and prometastatic microRNAs [61]. Curcumin affects the apoptosis of different cancer types through microRNAs. [58]. However, few clinical trials have shown the role of curcumin in altering DNA damage response by affecting microRNAs [58].

Hypoxia is a phenomenon that may provoke the development of various cancers. The expression of several microRNAs has been reported to be affected in response to hypoxia [62]. miR-21 and miR-210 are hypoxia-mediated oncogenic microRNAs (47,48), with demonstrated overexpression in urogenital cancers such as prostate and cervical cancers. In prostate cancer cells, curcumin downregulates miR-21, leading to an increase in phosphatase and tensin homolog gene (PTEN) and programmed cell death protein 4 gene (PDCD4) (target genes for miR-21), and a decrease in proliferation markers such as cyclin D1 [29]. In cervical cancer cells (SiHa HPV16-positive cells), curcumin downregulates miR-21, resulting in increased PTEN expression and decreased activation of the signal transducer and activator of transcription 3 gene (STAT3), which acts as a promoter of oncogenesis. [23]. However, another study in the CaSki HPV-16-positive cell line indicated no significant decrease in miR-21 expression after adding 80 *μ*M of curcumin at 48 h [22]. In two types of ovarian cell lines (PA1, A2780), curcumin induces apoptosis and suppresses tumor growth through the miR-21/PTEN pathway [42].

The cluster of miR-143/miR-145 was reported to have tumor suppressor effects in several cancers including prostate cancer [63]. However, there are few reports related to the upregulation of miR-143/miR-145 in pancreatic ductal adenocarcinoma and hepatocellular carcinoma [64, 65]. Curcumin significantly upregulates the miR-143/miR-145 in prostate cancer cells in a dose-dependent manner [34]. miR-145 directly suppresses octamer–binding transcription factor 4 gene (OCT4). It is an important gene in prostate cancer carcinogenesis, even in the drug-resistant phenotypes [66]. miR-143 suppresses phosphoglycerate kinase-1 (PGK1), which is associated with the progression of prostate cancer [36]. Moreover, miR-143 downregulates the autophagy-related 2B gene (ATG2B), which makes prostate cancer cells more vulnerable to radiation [34].

Although curcumin demonstrates great anticancer effects, it has a rapid metabolism, and its bioavailability is low. Therefore, its effects in human studies may not be the same in in vitro studies. Thus, many analogs including EF24, CDF, and desmethoxycurcumin (DMC) have been developed [67, 68]. EF24 significantly downregulates miR-21 in prostate cancer cells, which causes an increase in PTEN and PDCD4 expression [29]. CDF has been shown to decrease hypoxia-mediated microRNAs in prostate cancer cells [26]. DMC upregulated miR-551a in ovarian cancer cells, which suppressed the insulin receptor substrate 2 gene (IRS2) that acts as an oncogene in many solid tumors [27]. Nanotechnology approaches also have been used to increase the clinical utility of curcumin [69]. Yallapu et al. [28] assessed the efficacy of nanocurcumin (PLGA curcumin) in miR-21 expression in prostate cancer cells in comparison to curcumin. Although both significantly decrease miR-21, PLGA-curcumin showed more efficacy in cancer cell viability and proliferation.

Some studies demonstrated controversial results surrounding the impact of curcumin on microRNA expression in different cell lines. Wang et al. [46] showed that curcumin downregulates the expression of miR-7641 in bladder cancer cells, in opposition to Xu et al. [24]. Additionally, Ravindran et al. [42] showed controversial results regarding miR-122b, miR-129b, and miR-182 expression in different ovarian cancer cell lines.

While the majority of studies included in this review highlight a significant effect of curcumin on preventing the proliferation, development, and invasion of urogenital cancer cells, the outcomes of clinical trials are not promising. A Phase II randomized clinical trial (RCT) on patients suffering from metastatic prostate cancer indicated adding curcumin to docetaxel (an antineoplastic agent) did not significantly change the response rate, survival, and quality of life [70]. Another Phase II RCT on 64 cases of prostate cancer suggested the same results [71]. A study on 26 cases of muscle–invasive bladder cancer also demonstrated no significant change in the clinical response [72]. Although there is limited data on the clinical efficacy of curcumin in urogenital cancers, the mentioned studies highlighted the gap between basic research and clinical practice. Further RCTs on more patients with different stages of cancer may better clarify the clinical efficacy of curcumin.

This systematic review has some strengths. It represents the first systematic review on urogenital cancers, despite the plethora of published papers investigating the impact of curcumin on cancers via microRNAs. Additionally, our review delves into the effect of curcumin and its analogs on urogenital cancers through the expression of microRNAs. This systematic review has some limitations. Due to inadequate data availability, we were unable to conduct a meta-analysis. Furthermore, there is no suitable tool for the quality assessment of in vitro studies.

## 5. Conclusion

It can be concluded that curcumin and its analogs inhibit cell proliferation, development, and invasion and induce cell apoptosis in different urogenital cancers including prostate, bladder, ovary, kidney, and cervix cancers, through suppressing oncogenic microRNAs or provoking tumor suppressor microRNAs. However, controversies between the results of basic studies and clinical trials necessitate more clinical investigations for better clinical utility of the anticancer effects of curcumin.

## Figures and Tables

**Table 1:** Characteristics of included studies in the systematic review.

**Author and year**	**Country**	**Type of cancer**	**Cell type**	**Type of curcumin**	**Vehicle control**	**Endogenous control**	**miRNAs and outcome**	**Mechanisms of signaling**	**Comments**
Bao et al. (2012) [26]	USA	Prostate cancer	PC-3	CDF	NR	RNU48	miR-Let-7c, upregulation	NR	CDF could affect the expression of hypoxia-associated miRNAs in cancer stem cells of the prostate
							miR-Let-7d, upregulation	NR	
							miR-101, upregulation	NR	
							miR-210, downregulation	VEGF, CAIX, and HIF-1*α*-dependent mechanism	
Cao et al. (2017) [36]	China	Prostate cancer	DU145	CUR	DMSO	NR	miR-143, upregulation	Suppressing PGK1 and upregulating FOXD3	Inhibiting cell proliferation and migration
Du et al. (2017) [27]	China	Ovarian cancer	SKOV3	DMC	DMSO	U6	miR-551a, upregulation	Suppressing IRS2	Inhibiting cell proliferation and inducing apoptosis
Guo et al. (2020) [44]	China	Cervical cancer	HeLa, SiHa	CUR	DMSO	U6	miR-29b, upregulation	Suppressing PI3K/AKT axis via KDM2A	Inhibition of proliferation, migration, and invasion
Jianbo Liu et al. (2017) [34]	China	Prostate cancer	PC-3, DU145, LNCaP	CUR	NR	RNU48	miR-143, upregulation in a dose-dependent manner	Downregulating ATG2B	Inducing apoptosis and radiosensitivity of cancer cells
							miR-145, upregulation in a dose-dependent manner	NR	
Liu Te et al. (2017) [38]	China	Prostate cancer	CD44+/CD133+ HuPCaSCs	CUR	DMSO	NR	miR-145, miR-3127, miR-3178, miR-1275, miR-3198, miR-1908 upregulated	miR-145 suppresses Oct4, lncRNA-ROR	Suppressing proliferation and invasion
							miR-494, miR-193b, miR-671, miR-210, miR-222, miR-23b, miR-664, miR-183 downregulated	NR	
Saini et al. (2011) [45]	USA	Bladder cancer	T24, J82, and TCCSUP	CUR	DMSO	RNU48	miR-203, upregulated	Downregulating Src-Akt Axis	Decreased proliferation and increased apoptosis
Shishodia et al. (2014) [23]	India	Cervical cancer (HPV-16 positive)	SiHa (HPV16-positive)	CUR	DMSO	U6	miR-21, downregulated	Decreasing STAT3 and increasing PTEN	Inducing apoptosis
Sun et al. (2019) [47]	China	Renal cell carcinoma	ACHN, Caki-2	CUR	NR	U6	miR-106b, downregulation	XIST/miR-106b/P21	Inhibiting proliferation
Wang et al. (2018) [46]	China	Bladder cancer	T24, J82, and TCCSUP	CUR	DMSO	NR	miR-7641, downregulation	Upregulating P16	Inhibiting invasion and increasing apoptosis
Xu et al. (2019) [24]	China	Bladder cancer	T24, HT-1376	CUR	DMSO	NR	miR-1246, downregulation	Inhibiting P53	Inhibiting colony formation and inducing cell apoptosis
							miR-7641, miR-7110, miR-let-7c upregulation	NR	
Yallapu et al. (2014) [28]	USA	Prostate cancer	C4-2, DU145	CUR	DMSO	18S-rRNA	miR-21, downregulation	NR	Inhibiting tumor growth and inhibiting apoptosis. Moreover, PLGA-CUR had more efficacy than CUR.
				Nanocurcumin (PLGA-curcumin)	PLGA				
Yang et al. (2013) [29]	USA	Prostate cancer	DU145	EF24	DMSO	U6	miR-21, downregulation	Increasing the expression of PTEN and PDCD4	Inducing apoptosis
							miR-100, miR-126, miR-181a, miR-200a, no significant change		
Zhang et al. (2017) [43]	China	Ovarian cancer	A2780cp	CUR	DMSO	U6	miR-214, downregulation	PTEN/Akt pathway, and p53/Nanog axis	Inducing apoptosis
Zhang et al. (2018) [37]	China	Prostate cancer	DU145, 22RV1	CUR	DMSO	18S-rRNA	miR-770-5p, miR-1247, miR-411 upregulation	NR	Inhibiting cell proliferation and invasion
							miR-382, miR-654-3p downregulation		
Zhao et al. (2014) [39]	China	Ovarian cancer	SKOV3	CUR	DMSO	U6	miR-9, upregulation	Inhibiting Akt/FOXO1 axis	Inhibiting proliferation and inducing apoptosis in a dose-dependent manner
Zhao et al. (2017) [40]	China	Ovarian cancer	SKOV3	CUR	DMSO	U6	miR-124, not significant	NA	NA
Zhu et al. (2019) [30]	China	Prostate cancer	22RV1, PC-3, DU145	CUR	DMSO	U6	miR-34a, upregulation	Activating *β*-catenin/c-myc axis	Antiproliferation in a dose-dependent manner
Khojaste and Ahmadizadeh (2021) [22]	Iran	Cervical cancer	Ca Ski (HPV-16 positive)	CUR	DMSO	U6	miR-21, miR-210 not significant	NR	Inhibiting proliferation and inducing apoptosis
							miR-126 upregulation		
Pan et al. (2021) [31]	China	Prostate cancer	PC-3, DU145	CUR	NR	U6	miR-30a-5p, upregulation	Inhibiting PCLAF	Inhibiting proliferation, migration, invasion, and inducing apoptosis
Panahizadeh et al. (2022) [32]	Iran	Prostate cancer	PC-3 (CD44 positive and negative)	CUR	DMSO	U6	miR-383 upregulation	Downregulating LDHA and PRDX3	Suppressing proliferation and inducing apoptosis
							miR-708 upregulation	Downregulating RAP1B and LSD1	
Sun and Fang (2021) [41]	China	Ovarian cancer	SKOV3, A2780	CUR	DMSO	U6 and GAPDH	miR-320a, downregulation	Upregulating SMG1	Suppressing proliferation and inducing apoptosis
Vatankhah et al. (2022) [33]	Iran	Prostate cancer	PC-3	CUR	DMSO	NR	miR-148a, not significant	NA	NA
Ravindran et al. (2023) [42]	India	Ovarian cancer	PA1	CUR	DMSO	NR	miR-34a, miR-182, miR-199a, miR-335, miR-503 upregulation	Downregulating DDR1 through miR-199a	Inhibiting proliferation, migration, and invasion, and inducing apoptosis
							miR-21, miR-23a, miR-30a, miR-122b, miR-129, miR-320d, miR-1285 downregulation		
			A2780				miR-10b, miR-34a, miR-122b, miR-129 upregulation		
							miR-21, miR-30a, miR-32, miR-181a, miR-182, miR-205, miR-216a downregulation		
Li et al. (2024) [35]	China	Prostate cancer	PC-3, LNCaP	CUR	DMSO	U6	miR-483-3p, upregulation	Inhibiting UBE2C expression	Inhibiting proliferation, migration, and invasion

Abbreviations: CDF: 3, 4-difluorobenzylidene curcumin, CUR: curcumin, DMC: desmethoxycurcumin, DMSO: dimethyl sulfoxide, NR: not reported, PLGA: poly lactic co glycolic acid.

## Data Availability

The data that support the findings of this study are available from the corresponding author, upon reasonable request.
